# Elytral thickness and hidden species-specific traits shape passive thermal responses in beetles

**DOI:** 10.1242/jeb.252224

**Published:** 2026-04-27

**Authors:** Jorge M. Lobo

**Affiliations:** Department of Biogeography and Global Change, Museo Nacional de Ciencias Naturales–CSIC, c/ José Gutiérrez Abascal 2, 28006 Madrid, Spain

**Keywords:** Thermoregulation, Body mass, Body warming, Heating rates, Thermal strategies, Solar radiation

## Abstract

Body temperature strongly constrains insect activity, performance and survival, yet the mechanisms governing heat gain under solar radiation remain incompletely understood. In ectothermic insects, passive thermal traits may modulate body heating independently of energetically costly behavioral or physiological thermoregulation. Passive thermal responses were experimentally quantified in 147 freshly dead beetle specimens belonging to 34 Coleoptera species exposed to simulated solar radiation. Three complementary descriptors of body warming were measured – initial heating rate (IHR), final heating rate (FHR) and time required to reach 40°C (T40) – capturing distinct phases of passive heat acquisition. Elytral thickness emerged as the strongest morphological predictor of heating dynamics, showing a consistent negative effect on both IHR and FHR and a positive effect on T40. Body size influenced only the initial heating phase and had no effect on thermal resistance during prolonged exposure, indicating a partial decoupling between body size and passive thermal performance, whereas elytral darkness appeared to have only a slight influence on FHR. Substantial interspecific variation persisted after controlling for morphology and air temperature, revealing species-specific passive thermal strategies likely driven by unmeasured structural or compositional properties of the exoskeleton. These findings identify elytral thickness as a key determinant of passive thermal resistance in beetles while demonstrating that passive heating responses cannot be explained by body size alone. The persistence of species-specific differences suggests that additional, currently unknown exoskeletal traits contribute to thermal performance, highlighting passive thermal architecture as an underappreciated axis of ecological differentiation and thermal adaptation in Coleoptera.

## Teaser

**Summary:** Experimental exposure to simulated solar radiation in a range of beetle species indicates that elytral thickness is a key determinant of passive thermal resistance, but additional unknown exoskeletal traits can be determinant.

## INTRODUCTION

Temperature is one of the most pervasive environmental factors shaping the physiology, behaviour and distribution of organisms (Angilletta, 2009). In insects, which are predominantly ectothermic, body temperature largely determines metabolic performance, activity schedules, reproductive success and survival, thereby influencing both individual fitness and broader ecological patterns (Chown and Nicolson, 2004). Maintaining body temperature within favourable limits can be achieved through a combination of mechanisms in insects (Harrison et al., 2012). Among these, behavioural adjustments – such as microhabitat selection or changes in activity timing – often constitute the primary means of avoiding thermal stress (Abram et al., 2017), although physiological mechanisms, including evaporative cooling or metabolic heat production, may also contribute to thermoregulation in some taxa (Lahondère, 2023). However, all of these active strategies are energetically costly, may increase predation risk, and are strongly context dependent.

Because insects rely primarily on external heat sources to regulate body temperature, exposure to solar radiation has long been recognised as a major driver of insect thermal ecology (Casey, 1988; Chown and Nicolson, 2004). In this context, traits that enable passive regulation of body temperature – without requiring behavioural or physiological activity and the associated energy expenditure – may represent critical determinants of thermal performance. Such traits influence heat gain and heat loss through their interaction with solar radiation, thereby reducing the energetic costs of active thermoregulatory mechanisms and allowing energy to be allocated to other fitness-related processes. These processes correspond to what has been described as ‘passive thermal physiology’ (Carrascal et al., 2017) or ‘passive thermoregulation’ (Ospina-Rozo et al., 2022). Both concepts are closely related to the notion of ‘operative temperature’ (Bakken, 1992), defined as the steady-state body temperature attained by an organism under specific environmental conditions when heat gains and heat losses are balanced in the absence of active physiological thermoregulation.

Among the potential mechanisms underlying passive thermoregulation, morphological traits such as body size and shape, cuticle colouration or melanism, and appendage dimensions may play a central role in modulating heat exchange between the insect body and its environment (Angilletta, 2009). Body size has traditionally been regarded as a key determinant of thermal inertia, with larger individuals generally expected to warm up and cool down more slowly than smaller ones (e.g. Amore et al., 2017; Pincebourde et al., 2021; Kleckova et al., 2023). More recently, additional structural features of the insect exoskeleton – particularly cuticle thickness, which directly affects thermal conductivity – have emerged as strong candidates for shaping heat acquisition and resistance to overheating (see Carrascal et al., 2017; Ospina-Rozo et al., 2022; Lobo, 2025). The thermal relevance of such exoskeletal traits may be especially pronounced in Coleoptera, the most diversified animal order on Earth (Moodley et al., 2022), in which the presence of a pair of hardened forewings (elytra) provides a robust and continuous body covering.

Beyond their well-established roles in mechanical protection, desiccation resistance and wing protection (Goczał and Beutel, 2023), beetle elytra may also function as heat-absorbing or insulating barriers that modulate heat transfer into the thorax (e.g. Stuart-Fox et al., 2017; Alves et al., 2018). Comparative studies have documented substantial interspecific variation in cuticle and elytral thickness among beetles (e.g. Evans and Sanson, 2005; Kojima et al., 2024), yet the functional significance of this variation remains poorly understood, particularly in relation to thermal ecology. Moreover, it remains unclear whether heat acquisition and thermal resistance during solar exposure are primarily constrained by body size, exoskeleton thickness, or other structural or compositional properties of the cuticle. Using an experimental approach, the present study examined passive thermal responses in beetles by quantifying heating dynamics in freshly dead specimens, thereby avoiding the confounding effects of behavioural or physiological thermoregulation. Passive thermal responses were measured in approximately 150 coleopteran specimens belonging to 34 species under simulated solar radiation, using three complementary descriptors of body warming that capture distinct phases of the heating process. These thermal descriptors were related to body mass, body pseudovolume and elytral thickness in order to assess the relative importance of these morphological predictors, quantify interspecific and intraspecific variation in passive heating responses, and identify contrasting passive thermal strategies among species. By doing so, this study aimed to clarify the functional role of the beetle exoskeleton in thermal ecology and to evaluate the extent to which passive thermoregulation may be decoupled from body size. Understanding these mechanisms is essential for predicting how beetle species may respond to increasing solar irradiance and more frequent temperature extremes under ongoing climate change, and for identifying species that differ markedly in their passive thermoregulatory strategies.

## MATERIALS AND METHODS

### Data origin

A total of 147 specimens belonging to 34 species from 12 Coleoptera families were collected to examine their temporal internal temperature responses to a radiance source under controlled experimental conditions (Table 1). The specimens were obtained between 2021 and 2025 from 27 localities across nine provinces in mainland Spain (Table S1). Each specimen was assigned to a diel activity category based on published natural history information and the author's own field experience. Species were therefore classified as either diurnal or crepuscular/nocturnal (Table 1) to assess whether their thermal responses are generally associated with exposure to solar radiation under natural conditions. Similarly, species were categorised according to elytral coloration as having either black or dark brown elytra, or light/coloured elytra (Table 1). This classification was used to examine whether darker coloration is associated with greater heat gain, as predicted by the thermal melanism hypothesis (e.g. Clusella-Trullas et al., 2007).

**Table 1.** Beetle species examined, corresponding Coleoptera families and number of specimens (*N*) analysed for each species

Species	Family	*N*	IHR (°C min^−1^)	FHR (°C min^−1^)	T40 (min)	BM (g)	*V* (mm^3^)	ET (mm)	DA	DE
*Bolbelasmus bocchus* (Erichson, 1841)	Bolboceratidae	2	3.09	0.25	54.9	0.31	2277	0.31	CN	Y
*Pterostichus cristatus* (Dufour, 1820)	Carabidae	6	2.73	0.32	30.3	0.84	8426	0.41	CN	Y
*Scarites occidentalis* Bedel, 1895	Carabidae	1	4.60	1.33	9.8	0.73	7668	0.30	CN	Y
*Prionus coriarius* (Linnaeus, 1758)	Cerambycidae	4	2.60	1.08	16.6	1.23	12,919	0.19	D	Y
*Protaetia oblonga* (Gory & Percheron, 1833)	Cetoniidae	1	1.75	0.14	25.0	0.55	6166	0.31	D	Y
*Cerathophyus hoffmannseggi* (Fairmaire, 1856)	Geotrupidae	1	4.29	1.49	36.6	1.37	14,591	0.33	CN	Y
*Chelotrupes momus* (Olivier, 1789)	Geotrupidae	2	4.40	0.16	36.1	0.47	4391	0.31	CN	Y
*Geotrupes ibericus* Baraud, 1958	Geotrupidae	7	2.34	0.78	27.6	0.84	9218	0.34	CN	Y
*Geotrupes mutator* (Marsham, 1802)	Geotrupidae	14	3.45	0.91	27.4	0.62	6629	0.29	CN	N
*Geotrupes stercorarius* (Linnaeus, 1758)	Geotrupidae	15	2.91	0.51	32.4	0.76	8169	0.31	CN	Y
*Sericotrupes niger* Marsham, 1802	Geotrupidae	5	9.74	0.72	19.7	0.33	5395	0.27	CN	Y
*Thorectes lusitanicus* Jekel, 1866	Geotrupidae	2	4.25	0.05	42.0	0.47	4434	0.48	D	Y
*Trypocopris pyrenaeus* (Charpentier, 1825)	Geotrupidae	5	2.29	0.06	148.4	0.42	3979	0.40	D	N
*Typhaeus typhoeus* (Linnaeus, 1758)	Geotrupidae	6	9.64	2.21	6.2	0.38	3560	0.25	CN	Y
*Dorcus parallelipipedus* (Linnaeus, 1758)	Lucanidae	6	4.80	1.16	29.2	0.48	4575	0.45	CN	Y
*Lucanus cervus* Linnaeus, 1758	Lucanidae	5	1.46	0.35	55.8	2.47	32,038	0.73	CN	Y
*Amphimallon solstitiale* (Linnaeus, 1758)	Melolonthidae	6	12.60	6.78	4.0	0.24	2623	0.14	CN	N
*Melolontha melolontha* (Linnaeus, 1758)	Melolonthidae	6	5.77	5.13	5.8	0.81	9822	0.23	CN	N
*Pyrochroa coccinea* (Linnaeus, 1761)	Pyrochroidae	1	3.15	4.33	28.2	0.24	8213	0.37	D	N
*Bubas bison* Linnaeus, 1767	Scarabaeidae	1	5.48	3.50	15.6	0.32	7006	0.23	CN	Y
*Bubas bubalus* (Olivier, 1811)	Scarabaeidae	6	5.73	0.13	39.3	0.48	5935	0.32	CN	Y
*Copris lunaris* (Linnaeus, 1758)	Scarabaeidae	3	7.02	1.42	9.7	0.79	7496	0.35	CN	Y
*Onitis belial* Fabricius, 1798	Scarabaeidae	5	10.34	4.85	7.2	0.86	12,125	0.31	CN	Y
*Scarabaeus cicatricosus* (Lucas, 1846)	Scarabaeidae	4	3.35	0.44	20.5	1.28	12,836	0.31	D	Y
*Scarabaeus sacer* (Linnaeus, 1758)	Scarabaeidae	7	4.10	1.76	20.7	2.40	31,362	0.46	CN	Y
*Nicrophorus interruptus* (Stephens, 1830)	Silphidae	1	4.43	0.11	28.8	0.16	1170	0.66	CN	N
*Akis elegans* Charpentier, 1825	Tenebrionidae	2	1.95	0.55	37.3	0.62	5353	1.55	CN	Y
*Akis genei* Solier, 1837	Tenebrionidae	7	5.11	0.45	26.3	0.50	4563	0.41	CN	Y
*Blaps hispanica* Laporte, 1840	Tenebrionidae	3	0.95	0.48	45.9	2.25	26,746	1.01	CN	Y
*Blaps lusitanica* Herbst, 1799	Tenebrionidae	3	1.44	0.20	49.7	1.81	20,018	0.76	CN	Y
*Pimelia castellana* Pérez Arcas, 1865	Tenebrionidae	1	1.48	0.45	79.3	0.29	6903	0.53	D	Y
*Pimelia manchega* (Lauffer, 1905)	Tenebrionidae	6	7.44	0.72	27.2	0.43	4182	0.64	D	Y
*Sepidium* sp.	Tenebrionidae	2	4.54	0.24	54.7	0.31	2324	0.46	D	N
*Trox perlatus* (Goeze, 1777)	Trogidae	1	5.12	3.08	15.8	0.19	914	0.22	CN	Y

IHR, FHR and T40 represent the mean values of the initial heating rate, final heating rate and the time required to reach an internal body temperature of 40°C, respectively. BM, *V* and ET indicate mean body mass, mean pseudovolume and mean elytral thickness. DA denotes the diel activity of each species (CN, crepuscular or nocturnal; D, diurnal). The DE column discriminates between specimens with black or dark brown elytra (Y) on their dorsal side, and those with light or other colours (N).

### Experimental settings

Freshly dead specimens were weighed immediately prior to the thermal experiments using a Shimadzu^®^ Tx423L balance (precision: 0.001 g) to determine body mass (BM). Body length (*l*), width (*w*) and height (*h*) were measured using an Olympus SC100 CMOS camera attached to an SZX10 stereomicroscope. Length was measured from the clypeus to the tip of the abdomen, width as the distance between the humeral calli, and height at the mesothorax, where the second pair of legs is inserted. A pseudovolume (*V*) was calculated for each specimen using the ellipsoid formula *V*=4/3(π*lwh*). Body mass and pseudovolume were strongly and positively correlated in the examined specimens (Pearson *r*=0.96, *P*<0.001). Elytra thickness (ET) was measured on the right elytron in its anterior third, close to the elytral suture. Elytral thickness showed a weak but statistically significant positive correlation with body mass (*r*=0.39, *P*<0.01) and body pseudovolume (*r*=0.35, *P*<0.01). All three morphological variables were subsequently used to assess their influence on the heating parameters obtained. All specimens used were checked to ensure that none lacked the hardness and melanisation typical of fully sclerotised individuals, thereby avoiding the inclusion of newly emerged specimens.

Specimens were euthanised by exposure to −15°C for 15 min. Once dead, each specimen was perforated in the anterior third of the thorax, at the junction with the right elytron near the humeral callus. A fine type-K thermocouple was inserted through this opening until its tip reached the centre of the prothorax. Specimens were then cooled in a refrigerator until their internal temperature reached 0°C, after which they were transferred to the experimental arena. Internal body temperature was recorded every 15 s using a Fluke 54 II B dual digital thermometer (accuracy: 0.05%). Recording began once internal temperature reached 5°C and continued until 40°C. The experimental arena consisted of a 75 W halogen neodymium lamp emitting a balanced daylight spectrum (200–1600 nm; from ultraviolet to near-infrared, including part of the short-wavelength infrared range) simulating natural solar radiation (Fig. S1). The lamp was positioned 20 cm above the specimen, which rested on an expanded polystyrene foam platform. Air temperature (*T*_a_) during each trial was monitored using a second thermocouple placed 1 cm to the right of the specimen.

**Fig. 1.**
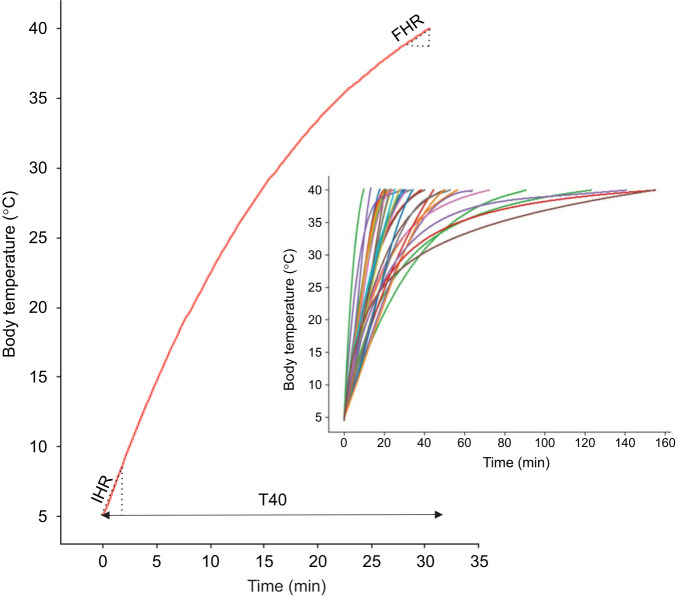
**Thermal parameters derived from curves representing the temporal increase in internal body temperature.** IHR: initial heating rate, defined as the rate of temperature increase during the first minute after reaching 5°C. FHR: final heating rate, defined as the rate of temperature increase during the final minute prior to reaching 40°C. T40: time required to reach an internal body temperature of 40°C. The inset shows examples of fitted functions illustrating the variability in the shape of the heating curves among the studied species.

The resulting heating curves, describing temporal increases in internal body temperature, displayed asymptotic or quasi-asymptotic shapes that varied among species shape (Fig. 1). To summarise this variation, three metrics were extracted from each curve (see Carrascal et al., 2017; Amore et al., 2017): (1) the initial heating rate (IHR), defined as the rate of temperature increase during the first minute after reaching 5°C; (2) the final heating rate (FHR), defined as the rate of temperature increase during the final minute prior to reaching 40°C; and (3) the time required to reach an internal body temperature of 40°C (T40). The lower and upper thresholds of 5°C and 40°C were selected because they approximate the minimum and maximum body temperatures at which insects typically enter torpor due to cold or heat (Vannier, 1994; Gallego et al., 2018). The IHR reflects the rate of warming in response to solar radiation during activation from cold stupor. Variation in T40 values indicates differences in the capacity to limit passive body heating in the absence of metabolic heat production; thus, dead specimens that require more time to reach 40°C are inferred to possess morphological or physical traits that reduce heat gain independently of active thermoregulatory mechanisms. Finally, the FHR reflects comparable properties related to thermal resistance as ambient temperature approaches the upper thermal tolerance limit (Weaving et al., 2022). These three parameters were only moderately correlated (Pearson *r*-values ranging from −0.46 to 0.54; see Fig. S2) and were therefore analysed separately.

### Statistical treatment

General linear models (GLMs) were fitted using all individual beetles to examine, separately, the relationships between each thermal response variable (IHR, FHR and T40) and the explanatory variables body mass (BM), body pseudovolume (*V*), elytral thickness (ET) and air temperature (*T*_a_). All predictors were standardised (mean=0, s.d.=1) to remove the effects of differing measurement scales. To account for potential non-linear relationships, quadratic polynomial terms were included for each predictor. Quadratic effects were considered statistically meaningful only when both the linear and quadratic terms were significant (*P*≤0.01). Additionally, a fully saturated model incorporating the quadratic functions of all four predictors was fitted to estimate the proportion of variability collectively explained by the full set of explanatory variables. The residuals from this saturated model represent the remaining unexplained variability for each thermal response variable. The first (25th percentile) and third (75th percentile) quartiles of these residuals were used to identify specimens exhibiting unusually low or high thermal values independently of their morphology.

GLMs with an analysis of covariance (ANCOVA) design were used to assess interspecific differences in IHR, FHR and T40 values, including the three morphological variables and air temperature during each experiment trial as covariates. Only species represented by five or more individuals were included in these analyses. In this context, the term ‘species’ refers to thermal-response variation among individuals belonging to the same taxonomic entity. Species identity was treated as a fixed factor, as the aim was not to characterise the full spectrum of thermal responses within Coleoptera, but rather to evaluate differences among the sampled ‘species’. Accordingly, any significant interspecific difference in one or more response variables were interpreted as reflecting species-specific thermal traits. The residuals from these models represent IHR, FHR and T40 values corrected for morphological constraints and ambient air temperature. The first (25th percentile) and third (75th percentile) quartiles of the adjusted mean values derived from GLM analyses – controlling for body morphology and air temperature – were used to identify species exhibiting unusually low or high heating rates and heating times. These species were considered potential candidates for possessing additional, unmeasured traits that may passively facilitate or constraint body heating.

Finally, morphology-corrected values of IHR, FHR and T40 were used to test for differences between diurnal and crepuscular/nocturnal specimens, as well as between specimens with black or dark-brown elytra and those with light or coloured elytra. These comparisons were performed using independent-samples *t*-tests, applying Welch's correction when variances between the two groups were unequal. Statistical analyses were performed in R (https://www.r-project.org/).

## RESULTS

### Overall variability in heating parameters

Heating parameters showed substantial variation both among and within species. The mean IHR across all specimens was 4.80°C min^−1^, although values were highly variable among individuals (s.d.=3.52, CV=73%, range=0.45–19.37°C min^−1^). Substantial intraspecific heterogeneity was also evident (e.g. *Geotrupes mutator*: s.d.=1.09, CV=32%, *N*=14; *Geotrupes stercorarius*: s.d.=1.82, CV=62%, *N*=15; see Table S1). The mean FHR across all specimens was 1.31°C min^−1^ and likewise showed high variability among specimens (s.d.=2.01, CV=154%, range=0.01–10.23°C min^−1^). The mean T40 was 31.0 min, again displaying marked inter-individual variation (s.d.=30.6, CV=98%). Considerable intraspecific dispersion was also observed for this variable in some cases (e.g. *Dorcus parallelipipedus*: s.d.=27.4, CV=94%). Variability in these three heating parameters was only slightly reduced when residual values from the saturated model – including morphological and air temperature as predictors – were considered instead of raw observations (s.d.=2.87, 1.82 and 28.54 for IHR, FHR and T40, respectively).

### Morphological determinants of heating

**Fig. 2.**
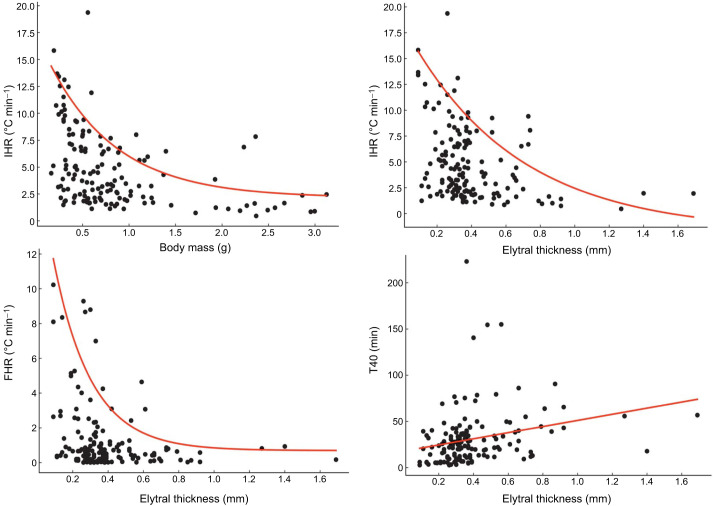
**Relationships between initial heating rate (IHR) and body mass, IHR and elytral thickness, final heating rate (FHR) and elytral thickness, and the time required to reach an internal body temperature of 40°C (T40) against elytral thickness.** The relationship between IHR and body pseudovolume is not shown, as it closely resembles that between IHR and body mass. Red lines represent negative exponential models fitted to the 95th quantile regression of the data, except for T40 versus elytral thickness, which was fitted with a linear model.

IHR was influenced by the morphological characteristics of the specimens. Body mass, elytral thickness and pseudovolume explained 22%, 14% and 13% of the total variability in the initial heating rate, respectively (Table 2). For all three morphological predictors, both linear and quadratic terms of the polynomial functions were statistically significant (*P*<0.001), indicating clear curvilinear relationships. These relationships revealed an upper constraint on heating performance. At smaller body sizes or with thinner elytra, IHR values were highly variable. However, as body size and elytral thickness increased, the maximum attainable heating rate progressively declined, following a decreasing exponential pattern that approached an asymptote of approximately 2.1°C min^−1^ in the largest specimens (Fig. 2). Specimens weighing more than 1.5 g and with elytra thicker than 0.75 mm showed no further reductions in IHR, suggesting a morphological threshold beyond which heating rates stabilise. A saturated model including quadratic functions of the three morphological variables together with air temperature explained approximately 33% of the total variability in IHR. The residuals of this model varied in both magnitude and direction among individuals within the same species. Negative residuals below the first quartile were recorded for only 15 individuals belonging to 10 species, whereas positive residuals above the third quartile were observed for 16 individuals across eight species. Notably, five of the six specimens of *Amphimallon solstitiale* and three of the five specimens of *Pimelia manchega* showed strongly positive residuals (see Table S1).

**Table 2.** General regression models showing the statistically significant relationships and the explanatory power of body mass (BM), body pseudovolume (*V*), elytral thickness (ET) and air temperature (*T*_a_) for the initial heating rate, the time required to reach an internal body temperature of 40°C, and the final heating rate

	Initial heating rate			Time to reach 40°C			Final heating rate		
	Function	*R*^2^×100	*F*	Function	*R*^2^×100	*F*	Function	*R*^2^×100	*F*
BM	Quadratic (– +)	21.76	20.03****		0.39	0.57		0.71	1.03
*V*	Quadratic (– +)	12.89	10.66****		0.15	0.22		0.11	0.17
ET	Quadratic (– +)	14.35	12.07***	Lineal (+)	6.38	9.88*	Quadratic (– +)	11.66	9.50***
*T*_a_		0.13	0.37		0.75	1.09		0.13	0.19
Saturated model		33.21	8.58****		12.77	2.52*		18.15	3.83**

The last row indicates the explanatory power of the fully saturated model incorporating the quadratic functions of all four predictors. A + sign in a linear function indicates a positive relationship, whereas quadratic functions are represented by two statistically significant terms; the – + signs indicate a U-shaped relationship. Significance levels are as follows: ****P<0.00001; ***P<0.0001; **P<0.001; *P<0.01.

In contrast to IHR, variation in FHR and T40 was explained almost exclusively by elytral thickness. This morphological variable is significantly associated with variation in FHR and T40, accounting for 12% and 6% of their total variability, respectively (Table 2). The relationship between FHR and elytral thickness was curvilinear, with both linear and quadratic terms statistically significant (*P*<0.001). Maximum FHR values followed a decreasing exponential trend: beetles with thinner elytra exhibited higher final heating rates, whereas those with thicker elytra reached an asymptotic plateau near 0.7°C min^−1^ (Fig. 2). In contrast, the relationship between T40 and elytral thickness was linear and positive (Table 2, Fig. 2), indicating that an increase of 1 mm in elytra thickness corresponded to an approximately 33 min increase in T40. A saturated model for FHR explained 18% of the total variability. Within this model, residuals were consistently negative and below the first quartile in 15 specimens belonging to nine species, with *Bubas bubalus* standing out, as three of its six examined specimens showed markedly negative residuals. Conversely, positive residuals above the third quartile were recorded for 15 specimens belonging to five species. In this case, all six individuals of *Melolontha melolontha* and four of the six specimens of *Amphimallon solstitiale* exhibited notably high positive residuals (Table S1). The saturated model for T40 accounted for 13% of the total variability. Negative residuals below the first quartile were observed in 16 individuals belonging to nine species, with *Pimelia manchega* standing out, as five of its six examined specimens showed such low residuals. In contrast, positive residuals above the third quartile were recorded for 15 specimens across nine species, most notably all six specimens of *Trypocopris pyrenaeus*, which exhibited high positive residuals (Table S1).

Including the qualitative variable representing elytra coloration in the previously mentioned saturated models with morphological predictors increased the total explained variability only slightly: from 33% to 34% for IHR, from 13% to 17% for T40, and from 18% to 24% for FHR.

### Influence of diel activity and elytral darkness

IHR residuals from the saturated model did not differ significantly between diurnal and crepuscular/nocturnal specimens (*t*=0.76, *P*=0.45), nor between specimens with dark and light/coloured elytra (*t*=1.15, *P*=0.25). FHR residual values were significantly lower in diurnal specimens (*t*=2.48, *P*=0.02) and in specimens with black or dark-brown elytra (*t*=2.25, *P*=0.03). In contrast, T40 residual values did not differ significantly between the two activity groups (*t*=1.90, *P*=0.069) or between the two categories defined by elytral coloration (*t*=1.63, *P*=0.11) (see Fig. S3).

### Species-specific differences

**Fig. 3.**
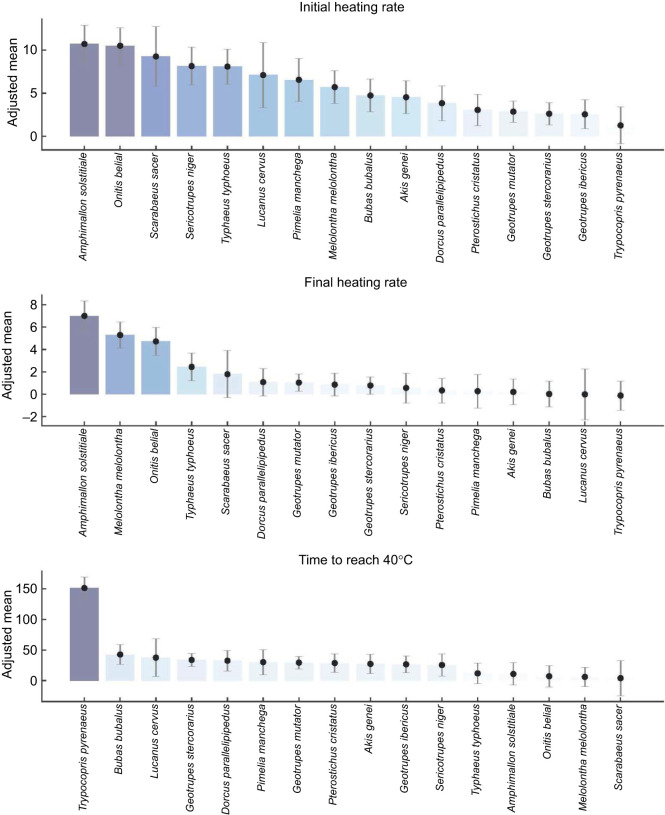
**Adjusted mean values (±95% confidence intervals) obtained from GLM analyses for each of the three response variables in the 16 species with five or more individuals (see Table 1)**. Models controlled for morphological variables (body mass, pseudovolume and elytral thickness) and for air temperature during the experiments. In each panel, species are arranged from highest to lowest according to their adjusted mean values.

**Fig. 4.**
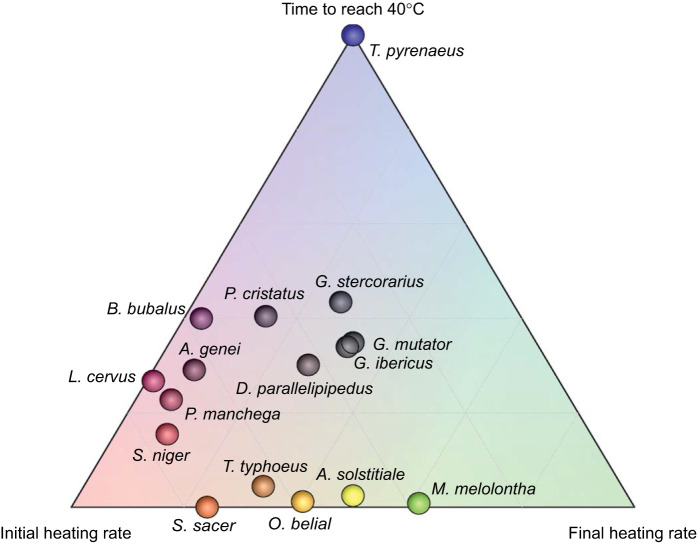
**Ternary plot showing the position of the 16 species with five or more individuals (see Table 1) within the space defined by the adjusted mean values obtained from the GLM analyses for the three response variables indicated at the vertices.** The closer to the bottom-left vertex, the higher the initial heating rate and the lower the other two variables. The closer to bottom-right vertex, the higher the final heating rate, whereas proximity to the upper vertex indicates a longer time to reach 40°C. Points located near the centre represent species with balanced values across all three variables.

When only species represented by five or more individuals were considered (16 out of 34 species; see Table 1), and air temperature and morphological variables were included as covariates, significant interspecific differences were detected for IHR (*F*_15,92_=10.02, *P*=0.0001), FHR (*F*_15,92_=11.44, *P*=0.0001) and T40 (*F*_15,92_=15.69, *P*=0.0001). *Amphimallon solstitiale*, and to a lesser extent *Onitis belial*, *Scarabaeus sacer*, *Melolontha melolontha* and *Typhaeus typhoeus*, exhibited higher IHR and FHR and reached 40°C more rapidly, independently of morphological effects (Fig. 3). In contrast, *Trypocopris pyrenaeus* displayed markedly slower heating rates and required substantially longer times to reach 40°C. When species were projected into the three-dimensional space defined by IHR, FHR, and T40 (Fig. 4), the distinctive thermal response profile of *T. pyrenaeus* became especially evident, clearly separating it from all other species and highlighting the existence of specie-specific thermal strategies that cannot be explained solely by morphology.

## DISCUSSION

The three thermal descriptors used in this study represent complementary phases of the body-warming process in the absence of active physiological thermoregulation and illustrate how morphological traits – particularly elytral thickness – modulate heat gain under experimental conditions simulating solar radiance. Taken together, these parameters describe key dimensions of a species' passive thermal architecture. The initial heating rate reflects how rapidly individuals can enter their daily activity window, the final heating rate captures their capacity to remain exposed to sustained solar radiation without excessive heat accumulation, and the time required to reach 40°C provides an integrated measure of thermal safety by defining tolerance to prolonged radiative exposure. Because these responses were measured in dead specimens, they reflect intrinsic structural and morphological properties rather than active physiological regulation. Variation in these parameters likely represents evolutionarily shaped differences in passive thermal performance, with direct implications for activity patterns, habitat use and vulnerability to thermal stress under natural conditions.

When exposed to a radiation source, IHR reflects the ability to convert incident radiation into body heat and warm up at the onset of activity (Carrascal et al., 2017; Amore et al., 2017). IHR therefore defines the speed at which an individual can enter its operative thermal window. From a biological perspective, high IHR values allow species or individuals to exploit short or unpredictable periods of favourable thermal conditions, potentially gaining early access to resources or reducing competitive interactions. Such rapid warming may be advantageous in cool environments or during early morning activity. Conversely, low IHR values indicate stronger passive thermal buffering, limiting heat gain under intense solar radiation. Variation in IHR values thus likely reflects an evolutionary trade-off between fast activation and protection against excessive heat gain.

The final heating describes how body temperature continues to increase following prolonged exposure to solar radiation (Carrascal et al., 2017; Amore et al., 2017). This parameter likely reflects the balance between ongoing energy absorption and simultaneous heat loss through conduction and convection. FHR therefore captures the ability of individuals or species to remain exposed to solar radiation once activity has commenced. Low FHR values indicate efficient passive heat dissipation and a tendency toward thermal quasi-equilibrium, where heat loss compensates for heat gain. Such individuals can remain active for longer periods without reaching critical temperatures, reducing the need for frequent sheltering and facilitating activity in open, sunny or highly isolated environments. In contrast, high FHR values suggest limited capacity for heat dissipation and an increased risk of overheating under sustained exposure.

The time required to reach an internal body temperature of 40°C provides an integrative measure of overall heat-gain efficiency under conditions lacking active thermoregulation. By incorporating the combined effects of both the initial and final heating phases, T40 reflects how quickly an individual approaches a critical or physiologically relevant thermal threshold. This parameter can be interpreted as a passive thermal safety margin, defining the duration for which an individual can tolerate radiative exposure before reaching potentially harmful temperatures. Short T40 values indicate high susceptibility to overheating and may be characteristic of species adapted to cooler or more thermally buffered environments. Conversely, long T40 values indicate greater resistance to solar radiation and enhanced thermal insulation. From an ecological perspective, individuals with longer T40 values possess a wider buffer against thermal stress, which may be critical in open habitats or under conditions of high solar irradiance.

### Variability in heating parameters

The results of this study indicate that high variability in all three heating parameters is the norm among coleopteran individuals and, in some cases, even among specimens belonging to the same species. Moreover, a substantial proportion of the observed variation remains unexplained, as it was only marginally reduced after accounting for morphological traits, air temperature and even elytral coloration. This pattern suggests that additional, unmeasured traits may play an important role in shaping passive thermal responses. Several factors not explicitly considered here may influence both heat gain and heat loss through convective and conductive exchanges with the surrounding environment. These may include physical properties of the exoskeleton, such as surface thermal conductivity (Galushko et al., 2005), microstructural organisation (Rivera et al., 2020), chemical composition (Reeves et al., 2021), optical properties (Jyoti and Tripathy, 2024), surface roughness, and degree of sclerotisation (Wang et al., 2019) and melanisation (Scalet et al., 2022). Internal traits, including the extent of the tracheal air-sac system (Verdú et al., 2012; Raś et al., 2024), body water content (Chown et al., 1998) and internal tissue density, may further modulate heat transfer. In addition, subtle factors such as slight differences in specimen orientation relative to the radiation source, body and elytra curvature, adult age, or variation in surface-area-to-mass ratios (Kühsel et al., 2017) may further modulate passive thermal responses. Taken together, these factors are likely to generate species-specific and individual-level passive thermal strategies that cannot be fully captured by external morphological measurements alone. This complexity may help explain the high residual variability observed in heating parameters and underscores the multifactorial nature of passive thermal performance in beetles.

### Body size and passive heating

Despite the limited ability of the selected morphological variables to explain a large proportion of the variability in passive thermal characteristics, body mass and pseudovolume had a significant effect on initial heating rates in the coleopteran specimens. Larger individuals consistently exhibited low initial heating rates and very little variation in these values, suggesting that large beetles are generally able to limit heat gain when exposed to solar radiation. In contrast, smaller specimens, characterised by a high surface-to-volume ratio, were capable of rapid heat gain but also included individuals that warmed much more slowly. This wide range of responses among small beetles likely reflects the combined influence of additional traits as those previously mentioned, which have not been captured by the external morphological measurements considered here.

Notably, neither the ability of beetles to remain exposed to solar radiation once activity has commenced, as measured by the final heating rate, nor the overall efficiency of heat gain measured by T40 were related to body mass or body volume. This indicates that passive thermal resistance during prolonged radiative exposure, as well as tolerance to critical temperature thresholds, are likely governed by mechanisms largely independent of body size. From an evolutionary perspective, this decoupling implies that resistance to prolonged radiative heating can be fine-tuned independently of overall body dimensions, allowing selection to act on passive thermal resistance without compromising size-dependent life-history traits. Consequently, beetle species of very different sizes may converge on similar thermal strategies when exposed to comparable environmental constraints, and both large and small species inhabiting similar thermal environments may evolve equivalent levels of thermal resistance through different, yet functionally convergent, mechanisms more closely linked to their thermal niche than to body size. Although body size is widely recognised as an important determinant of thermal inertia in ectothermic species (Pincebourde et al., 2021), several studies have shown that thermal tolerance does not necessarily arise as a direct consequence of body-size-related parameters (e.g. Verdú et al., 2006; Gunderson, 2024). Even in endothermic animals, thermal traits such as basal metabolic rate and thermal conductance may be largely independent of body size (Fristoe et al., 2015).

### The role of elytral thickness

Differences in cuticle thickness among closely related insect species have been documented previously (e.g. Peeters et al., 2017; van de Kamp and Greven et al., 2010; van de Kamp et al., 2016), although their biological significance has often remained unclear. The negative effect of elytral thickness on both initial and final heating rates observed here supports the view that beetle cuticle functions as an effective insulating barrier, delaying heat transfer into the body. Specimens with elytra thicker than approximately 0.7 mm never exhibited high heating rates, indicating that increased cuticular thickness enhances thermal stability and reduces the risk of overheating during prolonged radiative exposure. By contrast, individuals with thinner elytra displayed a wide range of thermal responses during both the initial and final phases of exposure to simulated solar radiation. Elytral thickness was also positively and linearly related to the time required to reach an internal body temperature of 40°C. Because this parameter represents a gradient from high susceptibility to overheating to increased resistance to solar radiation, this relationship indicates that individuals with thicker cuticles require substantially longer exposure to reach potentially stressful temperatures. Taken together, these results suggest that a thick beetle cuticle not only delays initial warming but also buffers heat accumulation as body temperature rises, thereby enhancing passive thermal resistance throughout the entire heating process.

The importance of exoskeleton thickness in insect thermoregulation has been previously proposed (e.g. Amore et al., 2017; Carrascal et al., 2017; Wang et al., 2021). However, the consistent influence of elytral thickness on all three thermal traits analysed here indicates that this morphological feature is likely a major determinant of passive thermal performance in beetles. Elytral thickness emerges as the single most consistent morphological predictor of passive thermal performance across all heating phases, being able to modulate both the rate and the extent of body heating and functioning as an efficient regulator of heat flow from the external surface into the thoracic cavity. Species with thicker elytra may therefore tolerate higher levels of solar radiation but may also experience delayed activity onset under cooler conditions, reflecting a thermal trade-off with potential ecological and behavioural consequences. At the community level, interspecific variation in elytral thickness may contribute to habitat filtering along thermal and radiative gradients, potentially influencing species turnover under increasing solar exposure and ongoing climate warming. Although body size did not emerge as a decisive determinant of thermal performance, cuticle thickness consistently constrained heat transfer more strongly than body volume or body mass. This pattern indicates a decoupling between body size and passive thermal resistance, suggesting that the efficiency of heat absorption and retention in the absence of active physiological regulation depends primarily on the structural and physical properties of the exoskeleton rather than on the total amount of body tissue to be warmed.

Elytral thickness thus represents a multifunctional trait shaped by interacting selective pressures, linking protection, desiccation resistance and other aspects of life-history components (Goczał and Beutel, 2023) with thermal ecology as a consequence of its key role in thermal insulation (Le et al., 2019; Ospina-Rozo et al., 2022; Goczał et al., 2025). Importantly, the apparent independence of thermal resistance from body size implies that passive thermal performance can be fine-tuned without altering overall body dimensions. This may allow species of very different sizes to converge on similar thermal strategies in response to comparable environmental constraints, while preserving other size-dependent life-history traits. As such, thermal adaptation in beetles may proceed less through changes in body size and more through modifications in exoskeletal design or composition, positioning cuticular traits as a prominent and previously underappreciated mechanism underlying the evolutionary responses of Coleoptera to thermal stress.

The relative importance attributed here to elytral thickness should nonetheless be interpreted with caution. Although Coleoptera exhibit an enormous range of body sizes – from less than 1 mg to more than 4 g (e.g. Ulrich, 2007) – the specimens analysed in this study spanned a more restricted range (0.16–3.12 g; see Table S1), partly excluding very small-bodied species for logistical reasons. In such taxa, size-related effects on heat exchange may be more pronounced. In addition, elytral thickness showed weak but statistically significant correlation with body mass and pseudovolume (*r*=0.39 and 0.35, respectively), indicating partial allometric dependence (see also Peeters et al., 2017). Consequently, it would be valuable to reassess whether some effects previously attributed to body size in Coleoptera (e.g. Tseng et al., 2018 or Fattorini et al., 2014) could be partially or wholly explained by variation in cuticular thickness. Finally, the mechanistic basis of the thermal insulation associated with increasing elytral thickness remains to be resolved. It is still unclear whether this effect arises from an overall increase in the thickness of one or more cuticular layers (Jafarpour et al., 2020; Muthukrishnan et al., 2022), or from structural or compositional modifications affecting thermal conductivity (Zhu et al., 2016; van de Kamp et al., 2016). The insect cuticle is a complex material composed of chitin–protein matrix containing lipids, phenolic compounds, minerals and water, arranged in layered microstructures that can vary substantially among species (Zhu et al., 2016). Future work should therefore aim to disentangle how variation in cuticle thickness, internal layering and material composition jointly determine thermal insulation, and how these properties have evolved in response to different thermal niches.

### Passive thermal strategies

Both the residuals of the saturated models using morphological variables as predictors and the analyses treating these variables as covariates indicate that, independently of the measured morphological traits, an interspecific gradient exists across the three heating parameters when freshly dead specimens lacking active thermoregulation are exposed to simulated solar radiation. Along this gradient, some species – such as *Amphimallon solstitiale*, *Onitis belial* and *Pimelia manchega* – exhibited a marked capacity for rapid body heating, whereas *Trypocopris pyrenaeus* clearly stands out for its exceptionally slow increase in body temperature. Species such as *Amphimallon solstitiale*, *Melolontha melolontha* and *Onitis belial* are also characterised by a limited ability to constrain internal body temperature during prolonged radiative exposure. In contrast, *T. pyrenaeus*, and to a lesser extent *Lucanus cervus* and *Bubas bubalus*, appear capable of remaining exposed to solar radiation without pronounced increases in body temperature. Overall, most of the examined species show a relatively high susceptibility to overheating, with *T. pyrenaeus* being the only species displaying a clearly higher resistance to simulated solar radiation.

These patterns allow the identification of contrasting passive thermal strategies that are not strictly dependent on body size or cuticle thickness. Some species can be classified as ‘heat-sensitive’, as they rapidly convert incident radiation into body heat (high IHR values), reach potentially harmful body temperatures quickly (low T40 values), and show limited resistance to heat accumulation under prolonged exposure (high FHR values). *Amphimallon solstitiale* is particularly representative of this strategy, although *Typhaeus typhoeus*, *Melolontha melolontha*, *Onitis belial* and *Scarabaeus sacer* exhibit broadly similar passive thermal profiles. At the opposite extreme, a ‘heat-resistant’ strategy is exemplified by *Trypocopris pyrenaeus*, which shows significantly slower initial and final heating rates and requires substantially more time than all other species to reach body temperatures associated with overheating. Rather than discrete categories, these strategies define a continuum of passive thermal responses probably shaped by evolutionary history, diel activity and habitat exposure. Within this continuum, species such as *Lucanus cervus* and *Geotrupes stercorarius* may also be regarded as relatively ‘heat-resistant’, albeit to a much lesser extent. Differences in life-history traits may help to explain these contrasting passive thermal behaviours. Notably, all species previously identified as ‘heat-sensitive’ exhibit crepuscular or nocturnal activity patterns, whereas the clearest example of a ‘heat-resistant’ species, *T. pyrenaeus*, is strictly diurnal (Zunino and Palestrini, 1986). *Trypocopris pyrenaeus* is also, to date, the only known geotrupid capable of tolerating a critical thermal maximum comparable to that of apterous species, which achieve such tolerance through evaporative cooling rather than passive insulation (Gallego et al., 2024). Statistically significant differences in heating parameters independent of morphology were detected only for the final heating rates, both between diurnal and crepuscular/nocturnal species and between species with black or dark-brown elytra and those with or light or coloured elytra. Thus, both diurnal species and those with dark elytra appear able to withstand solar radiation once activity has commenced, experiencing lower body heating rates than nocturnal or light-coloured species. Being dark in the visible light spectrum is not necessarily a trait associated with thermal regulation in insects (Turner and Lombard, 1990; Umbers et al., 2013; Yin et al., 2015; Lobo, 2024), and darkness has instead been linked to the absorption of the near-infrared fraction of solar radiation, which is invisible to the human eye (Cuesta and Lobo, 2021). In the present case, possessing dark elytra does not appear to increase body temperature and may even hinder warming, although its effect is weaker than that of elytral thickness. Further comparative morphological studies are required to identify which specific structural or compositional features of the exoskeleton underlie these interspecific differences in passive thermal performance.

Although the present experiments focused exclusively on heating dynamics under simulated solar radiation, the same morphological traits may also influence cooling processes once radiative input declines. Traits such as body size and cuticle thickness could affect the rate of heat dissipation through conduction and convection, potentially influencing afternoon cooling dynamics and the duration of activity periods in species exposed to intense solar radiation. Furthermore, it is necessary to recognise the probable link between the passive thermal processes examined here and behavioural thermoregulation. Both are likely to operate as complementary rather than independent components of insect thermal ecology. The interspecific passive thermal differences identified in this study may provide the structural background against which behavioural responses to solar radiation are expressed (e.g. Gardner et al., 2024). Species with limited passive thermal resistance may rely more strongly on behavioural mechanisms such as shade seeking, posture adjustment or temporal shifts in activity to avoid overheating, whereas species with more insulating exoskeletons may tolerate longer exposure and thus require less frequent behavioural compensation. In this sense, interspecific variation in behavioural thermoregulation may partly reflect underlying differences in passive thermal architecture. Finally, from a conservation perspective, increasing of solar irradiance and more frequent temperature extremes are likely to disproportionally affect species characterised by low passive thermal resistance, particularly where behavioural or physiological compensatory mechanisms are limited. By contrast, species possessing traits such as increased elytral thickness may exhibit a degree of structural pre-adaptive buffering against future thermal stress, potentially reducing their vulnerability under scenarios of climate warming.

### Conclusions

This study provides experimental evidence that passive thermal responses in beetles are shaped by a combination of morphological traits and species-specific properties that cannot be fully explained by body size alone. By isolating passive heating dynamics in the absence of behavioural and physiological thermoregulation, the results show that elytral thickness plays a central role in modulating both the rate and extent of body warming under simulated solar radiation. In contrast, body size primarily influences the initial phase of heating, whereas resistance to prolonged radiative exposure and tolerance to high body temperatures appear largely independent of overall body dimensions. Beyond morphological predictors and elytral darkness, clear interspecific differences in heating dynamics reveal the existence of contrasting passive thermal strategies among beetles, ranging from heat-sensitive to heat-resistant profiles. These strategies likely reflect evolutionary adjustments in exoskeletal structure and composition associated with the thermal niche of each species, rather than simple allometric constraints. The decoupling between body size and passive thermal resistance suggests that selection can act on exoskeletal traits to fine-tune thermal performance without compromising size-dependent life-history traits. These findings highlight passive thermal traits as key, yet often overlooked, components of insect thermal ecology. As solar irradiance and temperature extremes intensify under ongoing climate change, species with limited passive thermal resistance may face increased vulnerability, whereas those possessing structurally insulating exoskeletons may exhibit a degree of pre-adaptive buffering. Overall, this work emphasises the need to incorporate passive thermal architecture into trait-based frameworks to improve predictions of insect responses to thermal stress.

## Supplementary Material

10.1242/jexbio.252224_sup1 Supplementary information

Table S1. Raw data for all specimens included in the study, comprising the variables used as predictors or covariates: the three morphological variables and the air temperature recorded during each experimental assay. Initial heating rates (IHR; in °C min-1), final heating rates (FHR; in °C min^-1^), and the time required to reach an internal body temperature of 40 °C (T40; in min) are also reported, together with the residuals from the saturated models relating IHR, FHR, and T40 values to morphological variables and air temperature. The first (25th percentile) and third (75th percentile) quartiles of these residuals were used to identify specimens exhibiting unusually low or high heating rates and heating times. Positive and negative residuals are indicated by “+” and “-” symbols, respectively. Collection locality and collection date are also provided.
