# Supplementary material for: Elytral thickness and hidden species-specific traits shape passive thermal responses in beetles
Source: J Exp Biol. 2026 Apr 27;229(8):jeb252224. doi: 10.1242/jeb.252224 (PMC13200721; doi:10.1242/jeb.252224)
Supplement: Supplementary information [file jexbio-229-252224-s1.pdf]

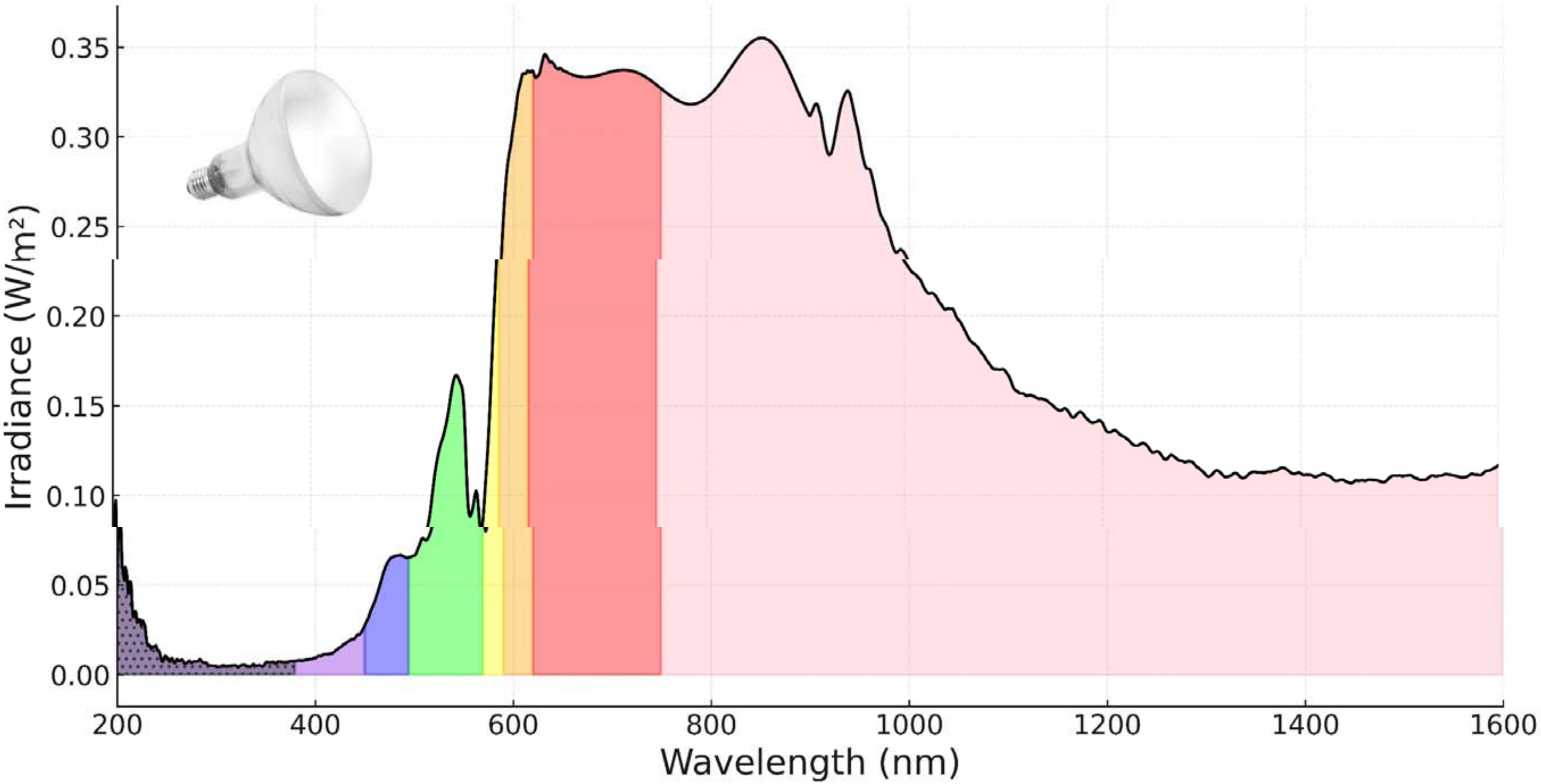

Fig. S1. Emission spectrum (from ultraviolet to near-infrared, including part of the short-wavelength infrared range) of the 75 W halogen neodymium lamp used in the experimental assays.

Journal of Experimental Biology • Supplementary information

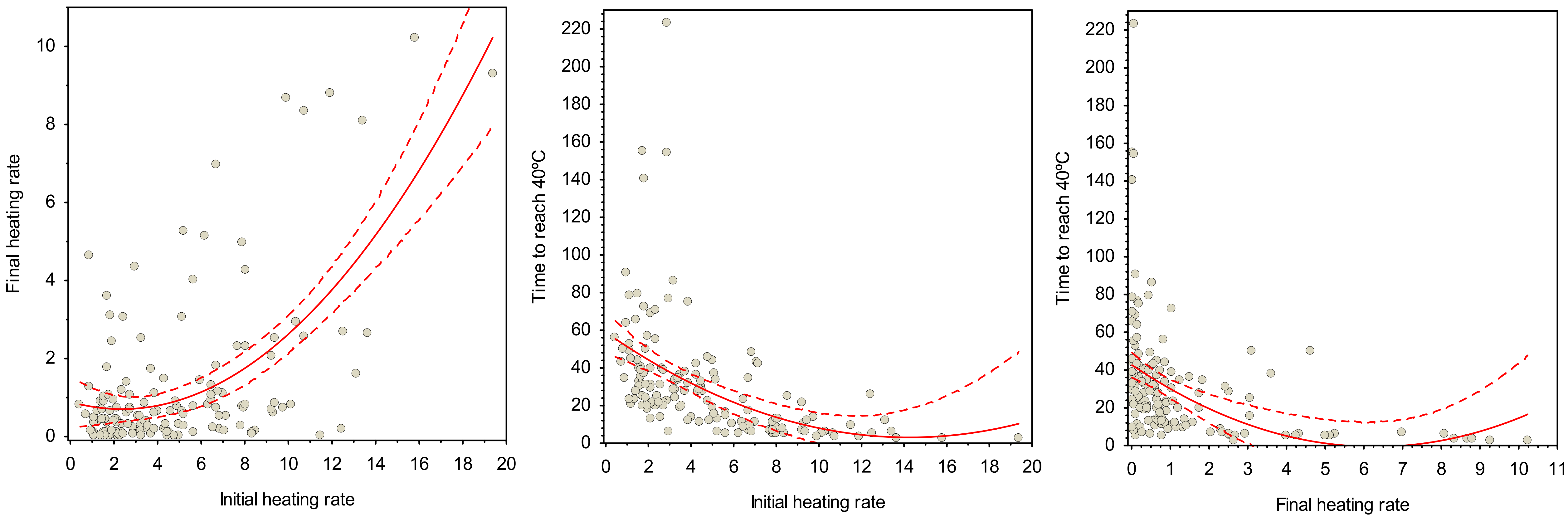

Fig. S2. Relationships between the initial heating rate and the final heating rate, between the initial heating rate and the time required to reach an internal body temperature of 40 ºC, and between the final heating rate and the time required to reach 40 ºC. Solid red lines represent fitted quadratic polynomial relationships, while dashed lines indicate 95% confidence intervals.

Journal of Experimental Biology • Supplementary information

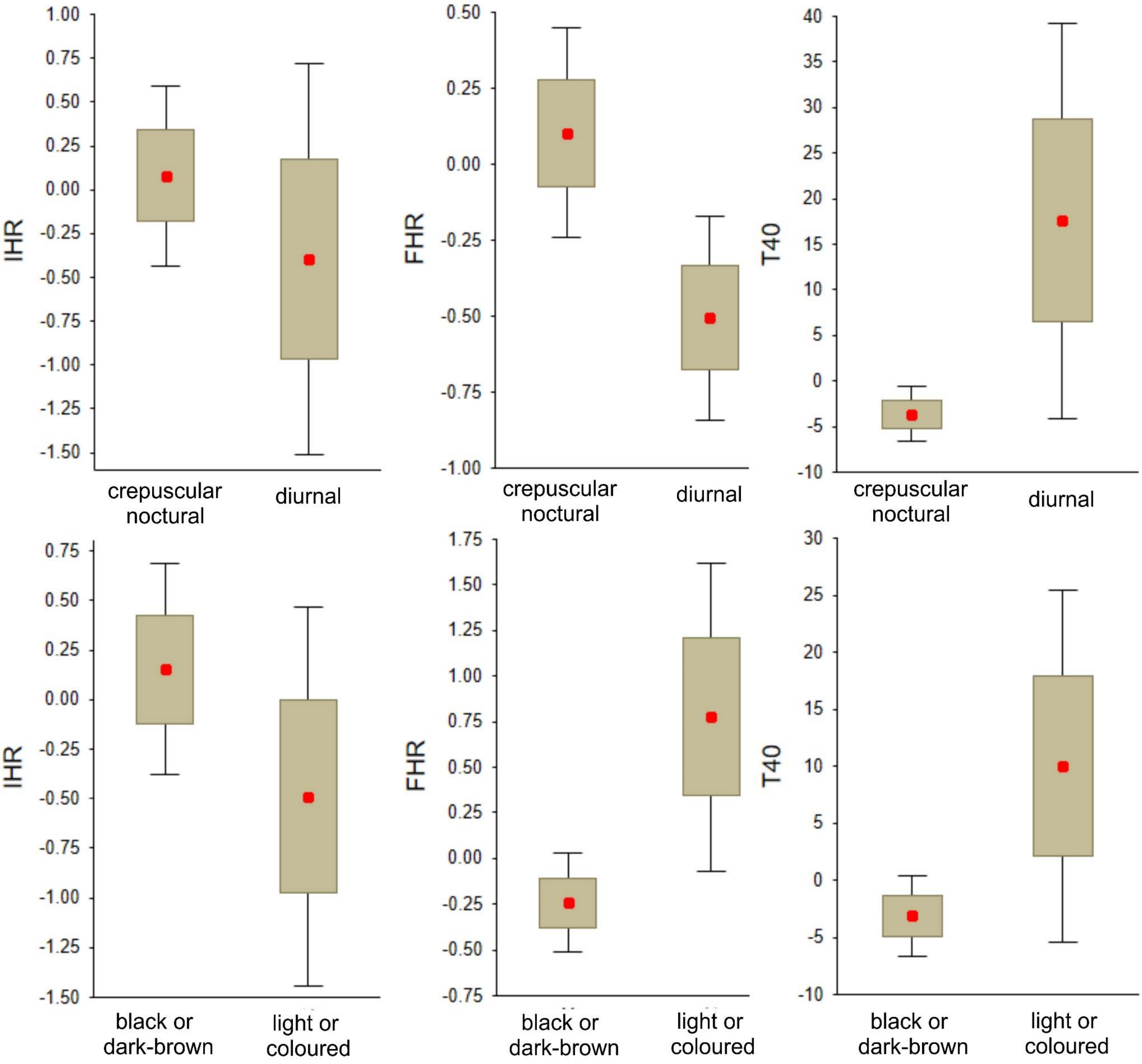

Fig. S3. Means (red circles), standard errors (boxes), and 95% confidence intervals (whiskers) of the morphology-corrected values of the initial heating rate (IHR), final heating rate (FHR), and the time required to reach an internal body temperature of 40 ºC (T40), according to diel activity (crepuscular/nocturnal vs. diurnal individuals) and elytral coloration (specimens with black or dark-brown elytra vs. those with light or coloured elytra).

Table S1. Raw data for all specimens included in the study, comprising the variables used as predictors or covariates: the three morphological variables and the air temperature recorded during each experimental assay. Initial heating rates (IHR; in ºC min^{-1}), final heating rates (FHR; in ºC min^{-1}), and the time required to reach an internal body temperature of 40 ºC (T40; in min) are also reported, together with the residuals from the saturated models relating IHR, FHR, and T40 values to morphological variables and air temperature. The first (25th percentile) and third (75th percentile) quartiles of these residuals were used to identify specimens exhibiting unusually low or high heating rates and heating times. Positive and negative residuals are indicated by "+" and "-" symbols, respectively. Collection locality and collection date are also provided.

Available for download at
https://journals.biologists.com/jeb/article-lookup/doi/10.1242/jeb.252224#supplementary-data
